# Balance chiropractic therapy for cervical spondylotic radiculopathy: study protocol for a randomized controlled trial

**DOI:** 10.1186/s13063-016-1644-2

**Published:** 2016-10-22

**Authors:** Feng Yang, Wen-xiong Li, Zhu Liu, Li Liu

**Affiliations:** 1 Shaanxi University of Chinese Medicine, Xi’an, 712000 China; 2 Affiliated Hospital of Shaanxi University of Chinese Medicine, Xi’an, 712083 China

**Keywords:** Cervical spondylotic radiculopathy, Balance chiropractic therapy, Efficacy, Safety

## Abstract

**Background:**

Cervical spondylosis is a very common disorder and cervical spondylotic radiculopathy (CSR) is the most common form of spinal degenerative disease. Its clinical manifestations focus on pain and numbness of the neck and arm as well as restricted movement of the neck, which greatly affect the patient’s life and work. The orthopedic of traditional Chinese medicine (TCM) theory holds that the basic pathologic change in spinal degenerative diseases is the imbalance between the dynamic system and the static system of the cervical spine. Based on this theory, some Chinese physicians have developed a balance chiropractic therapy (BCT) to treat CSR, which has been clinically examined for more than 50 years to effectively cure CSR. The purpose of this study is to evaluate the therapeutic effect and safety of BCT on CSR and to investigate the mechanism by which the efficacy is achieved.

**Methods/design:**

We propose a multicenter, parallel-group, randomized controlled trial to evaluate the efficacy and safety of BCT for CSR. Participants aged 18 to 65 years, who are in conformity with the diagnostic criteria of CSR and whose pain score on a Visual Analog Scale (VAS) is more than 4 points and less than 8 points, will be included and randomly allocated into two groups: a treatment group and a control group. Participants in the treatment group will be treated with BCT, while the control group will receive traction therapy (TT). The primary outcome is pain severity (measured with a VAS). Secondary outcomes will include cervical curvature (measured by the Borden Index), a composite of functional status (measured by the Neck Disability Index, NDI), patient health status (evaluated by the SF-36 health survey) and adverse events (AEs) as reported in the trial.

**Discussion:**

If BCT can relieve neck pain without adverse effects, it may be a novel strategy for the treatment of CSR. Furthermore, the mechanism of BCT for CSR will be partially elucidated.

**Trial registration:**

Clinical Trials.gov Identifier: NCT02705131. Registered on 9 March 2016.

## Background

Cervical spondylosis is a very common disorder and cervical spondylotic radiculopathy (CSR) is one of the most common patterns, accounting for about 60 to 70 % of all cervical spondylosis [1–3]. The incidence of CSR tends to increase year by year due to population aging, lifestyle changes and work or life stress. Its clinical manifestations focus on pain and numbness of the neck and arm as well as restricted movement of the neck, which greatly affect people’s lives and work. The agents currently approved for treatment and/or prevention of CSR include operative treatment and nonoperative treatment categories (including drugs, traction, manipulation, physical therapy, functional exercise, etc.), but many studies have confirmed that nonoperative therapy has more evident effects on the optimized scheme of CSR [4–6]. Among them, manipulation is the most common therapy for CSR due to its low risk, good therapeutic effect, ease of operation and its economics [7, 8]. However, as there are many kinds of manipulations with different efficacy standards, treatment and operation standardization and evaluation mechanisms, it is not conducive to the long-term development of massage, which could result in the problem of choice and multiple choices easy to cause medical information source waste. Moreover, there are currently few randomized, parallel-controlled trials to verify its efficacy in treating CSR.

From this we have developed a balance chiropractic therapy (BCT) to treat CSR, which has been clinically examined as effective at curing CSR for more than 50 years, and we examine the therapeutic effect and safety of BCT to improve the syndrome experienced by CSR patients in a multicenter, randomized, parallel-controlled trial. The results of this study will provide evidence regarding the value and safety of BCT as an intervention to improve the syndrome in CSR-affected individuals. Furthermore, we speculate the mechanisms of action can be partially identified by this study.

## Methods/design

### Study design

This clinical trial is a multicenter, parallel-group, randomized controlled design. Subjects will be enrolled at four hospitals: (1) The affiliated Hospital of Shaanxi University of TCM; (2) Shaanxi Provincial Hospital of TCM; (3) Xi’an Municipal Hospital of TCM; and (4) The Red Cross hospital of Xi’an.

### Ethical issues

This study has been approved by the Ethics Board of Shaanxi University of TCM (No: SZFYIEC-PJ-2016[01]). Each participating center obtained local Institutional Review Board approval. All study participants will sign the written informed consent prior to participation.

### Patients population and recruitment procedure

The study population consists of individuals aged 55 to75 years with CSR. The diagnostic criteria of CSR are referred to in the *Guidelines for the Diagnosis and Treatment of Cervical Spondylosis (2011 edition)* promulgated by the Chinese Association of Rehabilitation Medicine’s cervical spondylosis branch. The symptoms and signs include syndromes of pain and numbness distributing along spinal nerve roots and having positive intervertebral foramen extrusion and/or brachial plexus pull tests. Moreover, the clinical manifestations and imaging are consistent with the clinical syndromes.

Subjects will be excluded if they have disorders such as thoracic outlet syndrome, tennis elbow, carpal tunnel syndrome, cubital tunnel syndrome, periarthritis of the shoulder, tenonitis of biceps brachii, or a diagnosis of acute spinal cord injury, acute spinal cord inflammation, or symptoms of cervical vertigo and abnormal changes on transcranial Doppler (TCD). Subjects will also be excluded if they have associated pathologies of the liver, kidney, hematopoietic endocrine, cardiovascular or nervous systems and other severe primary diseases, or fractures, osteoarticular tuberculosis, osteomyelitis, bone tumor, severe osteoporosis, or mental disabilities, or other bodily weaknesses that cannot withstand the stimulation of BCT. Moreover, the trial will exclude individuals who have any acute infectious disease, gastric or duodenal ulcer with acute perforation, or treated areas of severe skin damage or skin diseases. In addition, subjects who have received surgical treatment for CSR or neck injury, or have received radiofrequency therapy to a cervical intervertebral disc, minimally invasive surgery, ozone, acupuncture and moxibustion, other manipulations or block therapy within 2 weeks, will also be excluded, as will lactating or pregnant female patients and patients who are participating in other clinical trials related to cervical spondylosis.

This study is to be conducted in accordance with patient protection principles as outlined in the Declaration of Helsinki, and approved by the appropriate Institutional Review Boards. Each participant will sign the written informed consent before undergoing any examination or study procedure in compliance with Good Clinical Practice. We will utilize a central randomization management system (CRMS) to identify all persons aged 18 to 65 years who meet the diagnostic criteria of CSR and who have a pain score on a Visual Analog Scale (VAS) of more than 4 points and less than 8 points. Patients who initially meet these eligibility criteria will then complete the additional baseline testing (mainly including a VAS, the Borden Index, the Neck Disability Index (NDI) and the 36-item Short Form health survey (SF-36) and will be randomized into either the treatment or the control group.

### Randomization and allocation

This clinical trial is a multicenter, randomized, parallel-controlled design. Randomization and sectionalization of subjects will occur centrally using a CRMS and patients will be randomly generated codes according to the treatment group or the control group in a 1:1 ratio. Potential participants will be identified via databases of four hospitals and then interviewed by phone for interest and eligibility.

**Fig. 1:**
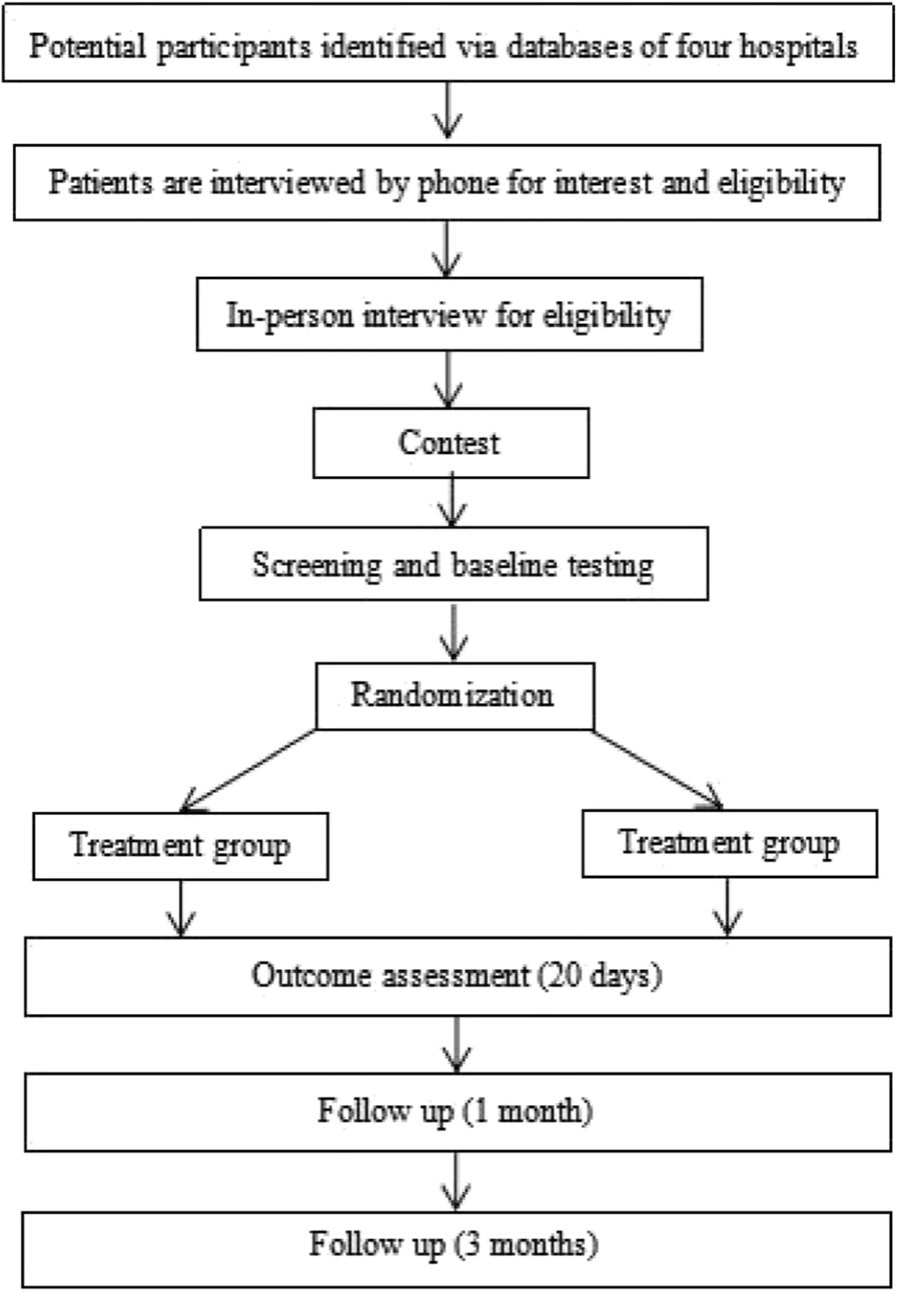
Flow diagram of recruitment process, group allocation and participation in the two interventions. All participants who completed a follow-up were included in the corresponding analysis

When the participants meet the inclusion or exclusion criteria and have signed the Informed Consent Form, researchers will access the CRMS and then input stratification factor according to the system’s prompt. The CRMS will display a participant identification code and a random number. The participant identification code or the random number is the only form of patient identity that distinguishes the treatment group from the control group. The flow of participants in the study, including the numbers analyzed for short-term and 6-month follow-up, are shown in Fig. 1.

### Interventions

Eligible patients will be randomized to one of the two arms: the treatment group and the control group. In the treatment group, patients will receive BCT for 20 days, while patients in the control group will receive traction therapy (TT) for 20 days. This study will be administrated at four hospitals. Patients will visit the physician at 1- and 3-month follow-up.

### Treatment group

In the treatment group, patients are requested to be in the sitting position and receive the following treatments: (1) balancing tendon-regulation: a to-and-fro kneading motion is applied three times to relax the muscles in the nuchal midline: splenius capitis, splenius cervicis and the trailing edge of sternocleidomastoid, respectively. Then manipulations of plucking and relaxing the tendons are applied five to seven times over the same area with a force that the patients can tolerate. Finally, rolling the region along the upper back of bladder meidian for five to seven times, (2) balancing osteopathy: firstly, with the patient adopting an upright sitting position, the practitioner holds the patient’s occiput and jaw between his hands and pulls upward forcefully for 9 s and then relaxes for 3 s. While stretching the neck, the physician turns the patient’s head to the front, back, left and right at an angle of roughly 45° three times and then obliquely wrenches the neck at the position that corresponds with the pathological features of the clinical examination and X-rays; if the lesion sites are at C1 to C3, or C4 to C6, or C7 to T1 within the cervical spine unit, the neck will be flexed at 15°, 0°, or 30 to 45°, respectively. The patient then repeatedly rotates their neck to left or right side to roughly 40° degrees on its own at the stretching state of cervical vertebra, and then rotates toward the affected side to the limit of the angle as well as bending the neck forward while the physician gives a vertical pulling and extending force to the patient’s neck. One or more snapping sounds will be heard if the procedure has been successful, (3) balance collaterals-dredging: first, holding the participant’s upper limb and then quickly shaking that upper limb up and down forcefully with a low-amplitude jittery motion, repeated three times. Next, for the ear-lifting method, kneading-pressing and pulling with the thumb and forefinger are then applied to the region of the upper, middle and lower three parts of the helix, respectively, for 30 s with a force that the patient can tolerate. Lastly, pressing with the thumb is repeatedly applied five to seven times along the DU meridian with focus on the acupoints DU4 (*Mingmen*), DU14 (*Dazhui*), DU17 (*Naohu*) and DU20 (*Baihui*).

The patients will received BCT once every other day for 20 min each session and five treatments constitute a course. The patients will be given two courses (a total of 10 times in 20 days) and will visit the physician at 1- and 3-month follow-up.

### Control group

In the control group, patients will received TT. The patient is sitting comfortably and wearing a cloth bag for occipital-jaw traction, with their head bending forwards at an angle of about 10–15°. The traction weight for cervical spondylosis starts at 3 kg and gradually increases to the maximum weight of 6 kg in increments of 0.5 kg each time. The treatment is performed 30 min at a time every other day for a total of 10 times in 20 days.

### Outcome measurements

The primary outcome is pain severity measured with a Visual Analog Scale (VAS) at baseline, 20 days of the treatment duration and 1- and 3-month follow-up. Secondary outcomes include cervical curvature measured using the Borden method, a composite of functional status measured by the Neck Disability Index (NDI), patient health status (evaluated by the SF-36 health survey) and adverse events (AEs) as reported in the trial.

### Assessment of pain

The VAS measures the amount of pain experienced and is a pain score ranging from 0 (no pain) to 100 mm (very severe pain). Operationally, the VAS score is usually a horizontal line, 100 mm in length, anchored by word descriptors at each end. The patient marks on the line the point that they feel represents their perception of their current pain. The VAS score is then determined by measuring in millimeters from the left hand end of the line to the point that the patient marks. The VAS score will be measured at all the measurement points (baseline, 20 days of the treatment duration, 1- and 3-month follow-up).

### The X-ray measurement of cervical curvature

**Fig. 2:**
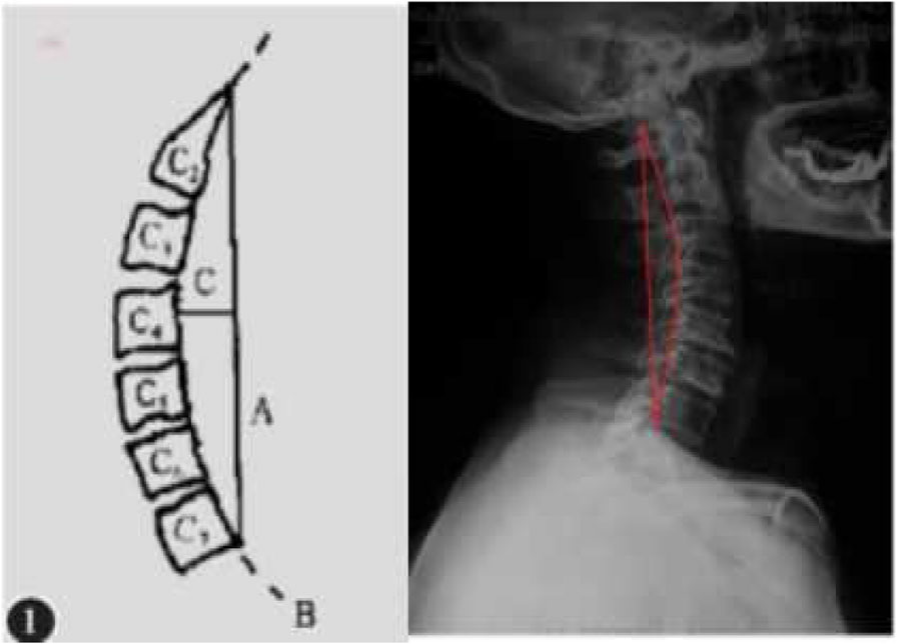
The schematic diagram for Borden’s method, which is a measure of the curvature of the cervical vertebrae

The schematic diagram for Borden’s method is shown in Fig. 2: in the lateral radiographs of the cervical vertebrae, line A runs between the posterosuperior margin of C2’s odontoid process and the posteroinferior margin of the C7 vertebra. A fitting curve along the posterior margin of the cervical vertebrae is line B. We define the vertical distance from the midpoint of C4 vertebra’s posterior margin to line A as the longest distance between lines A and B, which is taken as the curvature of the cervical vertebrae, line C) [9, 10].

### Neck Disability Index

As a measure of neck-specific functional disability, a translated version of the original 10-item Neck Disability Index (NDI) will be used [11]. The NDI covers 10 dimensions of neck-specific disability, namely pain intensity, personal care, lifting, reading, headache, concentration, work, driving, sleeping and recreation [11]. Each item assesses one dimension and is measured on a 6-point scale from 0 (no disability) to 5 (full disability). The overall score (out of 100) is obtained by adding the score for each item and multiplying by 2 [12]. A higher score indicates greater pain and disability [13].

### Assessment of patient health status

The SF-36 questionnaire was developed with the aim of measuring CSR patients’ health status. The SF-36 consists of 36 items, 35 of which are used in the calculation of eight separate scale scores. The physical functioning scale (10 items) is the longest scale. The general health and mental health scales have five items each and the vitality and role physical scales have four items each. The role emotional scale has three items and the bodily pain and social functioning scales have two items each. The remaining item of the SF-36 is a health transition question that asks about change in general health over the past 12 months [14]. This questionnaire will be completed at all the measurement points (baseline, the last day of the treatment, 1 and 3 months after the treatment).

### Assessment of adverse events

All subjects are to be questioned about AEs during treatment at each visit point and all those reported will be analyzed regardless of the investigators’ assessments of causality. The *Medical Dictionary for Regulatory Activities* (Med DRA, Version 8.1 J) will be used to categorize reported AEs.

### Sample size considerations

We calculated the sample size for this two-arm trial on the basis of comparing BCT versus TT, using the superiority test formula:

$$ {n}_1={n}_2=2{\left[\left(ta+t\beta \right)s/\delta \right]}^2, $$

where *tα* and *tβ* are constants, *s* is the estimated standard difference and *δ* is the mean value of the VAS for neck pain. The patients’ numbers were arranged in equal proportion and a superiority one-sided test was employed. Setting alpha at 0.05 and beta at 0.1, the calculated sample size of each group is around 98. Considering 20 % loss to follow-up, the total sample size needed to detect this difference at a 5 % level of significance with a power of 90 % is 240 patients [15, 16].

### Statistical analysis

The data will be collected and analyzed according to the intention-to-treat principle. Standard statistical techniques will be used to describe patients’ characteristics in both groups. We will compare baseline characteristics in both groups and, if incomparability appears, we will perform the secondary analysis, adjusting for differences. The primary outcome, VAS, will be compared between both groups using analysis of variance for repeated measures. If adjustment for possible baseline incomparability is needed, analysis of covariance will be done.

All data were analyzed statistically using SAS 9.2 version statistical software. Measurement data were expressed as mean ± standard deviation $\left(\tilde{x}\pm s\right)$, and analyzed using a *t* test; rates were compared using the chi-square test. *P* < 0.05 will be used to indicated that the difference was statistically significant.

## Discussion

CSR is one of the most common types of cervical disease. The orthopedic of TCM theory holds that both the static system (ligaments, joint capsules, etc.) and the dynamic system (muscles, intervertebral discs, small joints, etc.) are critical in maintaining normal position and function of the cervical spine. The imbalance of both static and dynamic forces can result in a loss of posterior column stability, ultimately leading to rapid degeneration of the cervical intervertebral discs and causing a series of syndromes distributing along the spinal nerve roots (such as pain, numbness of the neck, shoulder and arm, etc.) [17, 18]. Therefore, intervention measurements are aimed at correcting the imbalance between these two systems of the cervical vertebrae and relieving the pain which greatly affects people’s lives and work. For the determination of pain, the self-reported pain method is widely used and a VAS is an accurate method which is recognized internationally [19], hence our use of it for the primary outcome measure.

There is evidence that nonoperative treatment is more effective that operative for treating CSR [4–6], and tuina manipulation is the most common therapy with advantages such as low risk, good therapeutic effect, ease of operation and its economics. It has the effects of dredging meridians, stimulating *qi* and blood circulation, relaxing adhesions and correcting subluxation, ultimately correcting the vicious cycle of the imbalance between the dynamic system and the static cervical vertebral system and establishing the ‘virtuous circle’ instead which is beneficial to the rehabilitation of CSR and thus alleviates clinical symptoms [20–22].

Ears are the converging sites of the meridians and through the connection of meridians, ears are connected to the entire body. It is well-known that stimulating the aural helix can stimulate the corresponding meridians, so that regulating meridian *qi* and blood circulation, thus restoring *yin-yang* balance, will alleviate neck syndromes [23]. In addition, modern medicine shows that stimulating the auricular points can offset or weaken pain through a series of reactions [24]. In this study, BCT incorporates both tuina manipulation and stimulation of the auricular points.

If this study demonstrates the effectiveness and safety of BCT, significant strides would be made towards a clinically useful therapy for alleviating CSR syndrome.

### Trial status

The trial is still recruiting at the time of submission. The first participant was enrolled on 2 August 2016. The trial is expected to complete in August 2017.

## Abbreviations

AEs: Adverse events
BCT: Balance Chiropractic Therapy
CSR: Cervical spondylotic radiculopathy
NDI: Neck Disability Index
TCM: Traditional Chinese medicine
TT: Traction therapy
VAS: Visual Analog Scale
